# Heading Disorientation after Right Posteromedial Infarction

**DOI:** 10.1155/2015/396802

**Published:** 2015-10-07

**Authors:** Ritsuo Hashimoto, Noriyo Komori, Masako Abe

**Affiliations:** ^1^Department of Neurology, International University of Health and Welfare Hospital, 537-3 Iguchi, Nasushiobara, Tochigi, Japan; ^2^Department of Rehabilitation, International University of Health and Welfare Hospital, 537-3 Iguchi, Nasushiobara, Tochigi, Japan; ^3^Department of Speech and Hearing Sciences, International University of Health and Welfare, 2600-1 Kitakanemaru, Ohtahara, Tochigi, Japan

## Abstract

We report the case of heading disorientation following ischemic stroke involving the right posteromedial areas. The patient was administered a new test named the Card Placing Test during which a subject was required to recreate an array of three cards, each of which was randomly placed on eight grids around the subject, before and after the subject's rotation. Qualitative analysis of his performance after rotation revealed that over half of the errors comprised transposition and rotational offset. His score on the Card Placing Test was compared with those of normal controls (*n* = 11). The results showed that his score on Card Placing Test after rotation was significantly lower than those of controls, whereas there was no significant difference between the case and controls in profile of error types. We infer that the heading disorientation observed in the present case was a result of a derangement of a short-term buffer that integrated information on spatial locations of objects with changes in body directions.

## 1. Introduction

There is growing evidence that the posteromedial brain area including the retrosplenial cortex (RSC) plays a key role in a range of cognitive functions including spatial memory and navigation. Indeed, human neuropsychological studies have shown that patients with damage involving the RSC can demonstrate selective deficit in spatial orientation. The spatial disorientation seen in these patients is known as a type of pure topographical disorientation, namely, heading disorientation (HD).

In previous case studies, patients with HD were commonly evaluated by assessing their ability to recognize familiar landmarks, to ascertain spatial locations of immediate objects or landmarks, to draw maps of familiar places, and to describe routes. These assessments revealed that most of the patients were oriented within their immediate environment and can localize objects relative to their body (preserved egocentric orientation) but could not apprehend the relationship between the immediate environment and locations beyond it that were not currently visible (lost allocentric orientation) [1–3]. These findings are very suggestive; however, the tests used were qualitative in nature and the patients' performance is thought to have depended on how much knowledge they had about the routes or maps of the environment before their illness.

To solve these issues, we recently developed a clinical test named Card Placing Test (CPT) [4]. The CPT requires a subject to recreate an array of three cards, each of which was randomly placed on eight grids around the subject, before (CPT-A) and after the subject's rotation (CPT-B). With this design, the CPT can assess a subject's ability to represent visuospatial information either egocentrically (CPT-A) or allocentrically (CPT-B). We demonstrated that HD patients with damage involving the RSC showed good record results for CPT-A but very poor ones for CPT-B [4]. Thereafter, correlation mapping studies using SPECT [5] or PET [6] revealed that performance of the CPT-B was associated significantly with regional cerebral blood flow in bilateral posteromedial area(s) including the RSC. Taken together, it is suggested that the CPT is a useful clinical test to evaluate patients with HD and that CPT-B performance is associated with a function of the posteromedial area including the RSC. Then, we thought that some more detailed presentation of the behavioural data associated with the CPT performance would give more information. More specifically, if damage to the posteromedial area was specifically associated with a kind of error in the performance of the CPT, an analysis of that could reveal something interesting about the function of the posteromedial area.

Here we reported a patient who presented with HD after an infarction involving the right posteromedial area. We performed detailed neuropsychological examinations including the CPT. The performance of CPT was evaluated not only by the scores of the CPT but also by the types of errors that the patient demonstrated. We then argued over the nature of HD shown by the present case.

## 2. Case Presentation

A 74-year-old right-handed man, with 9 years of education and who had been under treatment for diabetes mellitus, hypertension, and atrial fibrillation for over 4 years, complained of sudden headache and blurred vision while on voluntary work. He initially thought that it might be due to hypoglycaemia and took glucose granules, which did not give symptomatic relief. He tried to go back home but it was not easy for him. Although he could identify buildings, trees, and roads that were familiar to him, he was unable to determine to which direction he should turn. After having made many wrong turns along the way, he eventually arrived at his house; it had taken him 2 h, compared with his usual 20-min walk. Three days later, he was referred to our hospital.

**Figure 1:**
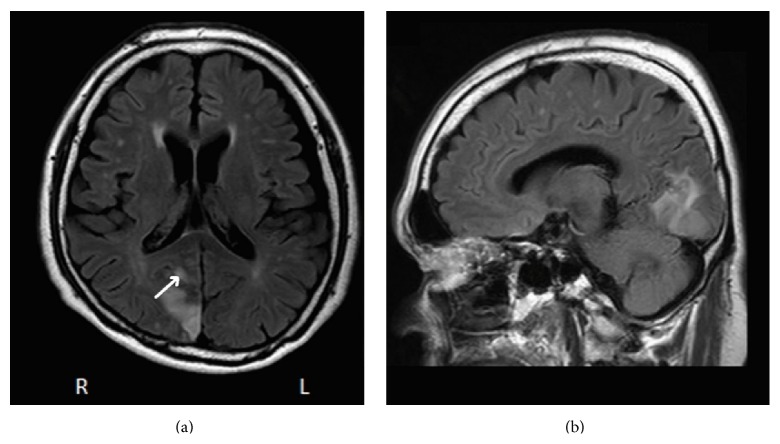
FLAIR MRI scan of the case on (a) axial and (b) sagittal views. High-signal intensity can be seen in the right posteromedial region that extended into the anterior bank of the parietooccipital sulcus (arrow). FLAIR: fluid attenuated inversion recovery; MRI: magnetic resonance imaging.

On admission, he had atrial fibrillation with no signs of heart failure. Neurological examination was normal except for a left homonymous hemianopia. Magnetic resonance imaging (MRI) on admission day disclosed a new infarct in the right posteromedial region (Figure 1). Magnetic resonance angiography (MRA) on the same day demonstrated occlusion at the P2 portion of the right occipital artery.

During his 1-month stay at our hospital, he demonstrated marked topographical disorientation. He was unable to go to the same rehabilitation room in our hospital unless someone instructed him each time he encountered a point that required him to turn.

### 2.1. Neuropsychological Evaluation

On evaluation, he was alert and cooperative. His Mini-Mental State Examination (MMSE) score was 28/30 and Raven's Coloured Matrix Test score was 32/36. His performance on Wechsler Adult Intelligence Scale-III was within normal range; he had a verbal intelligence quotient (VIQ) of 111, performance intelligence quotient (PIQ) of 97, and total intelligence quotient (TIQ) of 105. Thus, his global intellectual function seemed intact. As for memory function, his verbal memory quotient was 100 and that of visual memory was 82 on the Wechsler Memory Scale-Revised (WMS-R, Japanese version). On the Rey-Osterrieth Complex Figure Test (ROCFT), his scores for copying, immediate recall, and delayed recall 30 min after copying were 36/36, 18/36, and 18/36, respectively. With regard to visual function, he did not show any visual agnosia such as object agnosia or prosopagnosia. The line bisection and line cancellation tasks did not reveal hemispatial neglect. On Visual Perception Test for Agnosia (VPTA), only the topographical disorientation of his daily life was noted. He could easily identify pictures of landmarks near his house and could put each of those pictures at a precise location on a bird's eye view of his vicinity. He could draw a map of his town in detail; however, he showed great difficulties in depicting a map from his room to the rehabilitation room of our hospital (Table 1).

### 2.2. CPT

Two days after admission, we administered CPT [4], a new test that we developed involving the following: the patient was asked to stand in the centre of 3 × 3 grid of squares drawn on the floor with three cards randomly arrayed around him; after a study period, the cards were removed and the subject was asked to recreate the array while his standing position was unchanged (CPT-A) and after rotation (CPT-B). The subject underwent ten consecutive trials for CPT-A and CPT-B. He was scored one point each time he placed one card in the correct place. The maximum score for each part was 30 points. His scores for CPT-A and CPT-B were 28/30 and 14/30, respectively. Fourteen days later, CPT was repeated and scores for CPT-A and CPT-B were 30/30 and 18/30, respectively. Then, we analysed his errors qualitatively and classified those into three types: “transposition” error, wherein the patient placed a wrong card in a spatially correct grid; “rotational offset” error, wherein the patient placed two or three cards in wrong grids while maintaining their spatial relationship; and “uncategorized” error, wherein an error did not belong to the aforementioned ones. His errors in CPT-B on first examination comprised 10 transpositions, 2 rotational offsets, and 4 uncategorized errors; errors upon reexamination were 5 transpositions, 2 rotational offsets, and 5 uncategorized errors (Table 2).

### 2.3. Comparison with Controls

We conducted CPT in 11 healthy right-handed volunteers (4 men and 7 women) who had never experienced a brain disease such as cerebrovascular disease, head trauma, or epilepsy. Their ages ranged from 60 to 75 (68.3 ± 5.0 (mean ± SD)). Their MMSE scores ranged from 27 to 30 (29.0 ± 0.9 (mean ± SD)). They were independent in daily life and did not complain of topographical disorientation. All of them scored over 24/30 (28.2 ± 1.6 (mean ± SD)) in CPT-A and over 20/30 (23.5 ± 1.8 (mean ± SD)) in CPT-B. Control subjects demonstrated 69 errors on CPT-B performance in total. The errors comprised 33 transpositions, 11 rotational offsets, and 25 uncategorized errors. Statistical analysis revealed that his score for CPT-A was within normal range, whereas his score for CPT-B was significantly lower than those of controls (*χ* ^2^ test; *p* < 0.001). As for profile of error types on CPT-B performance, there was no significant difference between the case and controls (Table 3).

### 2.4. Follow-Up Study

Two months after the stroke, he was able to orient himself in a very familiar environment such as his neighbourhood. However, he still showed disorientation in a new environment.

## 3. Discussion

The present case showed topographical disorientation characterized by the following: (i) inability to derive directional information from landmarks despite his retained ability to recognize them; (ii) topographical disorientation in a new as well as a familiar environment; (iii) a mild anterograde visual memory disturbance; (iv) absence of apparent visual cognitive deficits, as demonstrated by no signs of hemispatial neglect, no signs of visual agnosia, and intact constructive abilities; and (v) fairly well-preserved general intellectual functions. Based on the taxonomy of topographical disorder proposed by Aguirre and D'Esposito [1], the present case seemed to be showing a type of pure topographical disorientation, that is, HD.

The retrosplenial and posterior cingulate cortices (RSC/PCC) have been suggested to be the responsible brain regions for development of HD [1–4]. The lesion in the present case, however, barely involved the RSC/PCC* per se*. Given the exact similarities in symptoms of the present case with previously reported cases of HD after RSC/PCC injury, a plausible explanation is that the ischemic lesion of the present case may have disrupted efferent and afferent subcortical fibers in RSC/PCC, causing a similar lesion effect to that of RSC/PCC. Another potential account is that the anterior bank of the parietooccipital sulcus, which is actually involved in the present case, may be a place that plays a pivotal role to provide the “sense of direction” in a large-scale locomotor environment [7].

We previously reported that patients with HD who had posteromedial lesions involving the RSC/PCC showed good results for CPT-A but very poor ones for CPT-B [4]. The present case reproduced this observation. The most intriguing findings of the present case were drawn from qualitative analyses of his errors in performing CPT-B. In his performance, there were fragments of encoded information about items and their spatial layout and self-rotation. In other words, over half of the errors were categorized as transposition (retained information about spatial layout and self-rotation but missing item information) or rotational offset (preserved information about items and their spatial layout but lost self-rotational information). Moreover, there was no significant difference between the case and normal controls in profile of error types on CPT-B performance suggesting that his cognitive dysfunctioning was not qualitative but rather quantitative in nature.

Overall, it is suggested that his errors stemmed from the disintegration of information on spatial layout of objects derived from an egocentric reference frame and on changes of his body direction. These phenomena may be explained by shrinkage of a short-term buffer for processing visuospatial information, which is mediated by the posteromedial area involving the RSC/PCC. In the present case, when he consumed the resource for processing self-rotational information and spatial layout, a decay of information about items occurred as the transposition error, and when he used his cognitive resource for retaining information about spatial layout and items, he became unable to attain self-rotational information that resulted as the rotational offset error.

In agreement with what was mentioned above, recent studies suggest that the posteromedial area involving the RSC/PCC functions as a connectional hub that gathers different types of information from the head directional system and medial temporal lobe memory system; it also interacts with the lateral parietofrontal network, which presumably contributes to the processing of information on egocentric visuospatial locations of objects [8–12]. Of note, Burgess and his colleagues have suggested a neural-level model of the medial temporal and parietal roles in retrieving spatial context of an event [13, 14]. Their assumption is that medial temporal allocentric representations are used in long-term storage, whereas parietal egocentric representations are used to imagine, manipulate, and reexperience the products of retrieval, with the RSC/PCC translating between the two codes.

The poor performance in CPT-B in the present case may be interpreted as a translation defect from the egocentric representation to an allocentric one following rotation. Given observations that the present case could easily identify pictures of landmarks near his house and could put each of those pictures at a precise location on a bird's eye view of his vicinity, his disorientation in a familiar environment could be a result of the difficulty in translating the stored allocentric map of the environment in order to guide actions according to an egocentric perspective [15].

In conclusion, the results of the CPT in the present case provide evidence that the posteromedial area involving the RSC/PCC operates as a short-term buffer that integrates information on the spatial locations of objects derived from an egocentric reference frame with that on changes of body directions. The buffer might function to translate egocentric representations of visuospatial information into allocentric ones and vice versa. The derangement of the system could result in HD as shown in the present case.

## Figures and Tables

**Table 1:** Neuropsychological test results in the present case.

*General cognition ability*	
MMSE (/30)	28
WAIS-III	
Total IQ	105
Verbal IQ	111
Performance IQ	97
Verbal comprehension	111
Sensory integration	97
Working memory	109
Processing speed	94
*Memory*	
Wechsler Memory Scale-R	
Verbal memory	100
Visual memory	82
General memory	94
Attention/concentration	89
Delayed recall	85
RBMT	
Standard profile (/24)	19
Screening profile (/12)	9
Rey-Osterrieth Complex Figure Test	
Copy (/36)	36
Immediate (/36)	18
Delayed recall (/36)	18
*Visual function*	
Visual Perception Test for Agnosia (VPTA)^*∗*^	
Basic visual function (/36)	1
Object/figure cognition (/26)	0
Facial cognition (/68)	1
Colour cognition (/78)	0
Symbol cognition (/74)	0
Visuospatial cognition and its manipulation (/228)	0
Topographical orientation	
Daily life (/6)	4
Autobiographical topographical knowledge (/4)	0
Allocation of prefectures and landmarks on	0
a map of Japanese islands (/16)	
*Map drawing*	
Mapping his familiar place	Preserved
Mapping his new environment	Disturbed
Allocating landmarks of the patient's hometown on a map	Preserved

MMSE: Mini-Mental State Examination; RBMT: Rivermead Behavioral Memory Test. ^*∗*^On VPTA, if there is a mistake or failure in performance, each of them will be scored 1 or 2 points according to the criteria; 0 means that there is no disturbance.

**Table 2:** Results of CPT-B on the present case.

Trial number	Primary position		First examination (2 days after admission)		Second examination (16 days after admission)	
			After rotation	Error type	After rotation	Error type
1	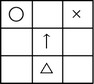	$\overset{{\text{90}}^{{}^{\circ}}\text{ to the right}}{\to }$	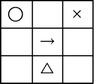	—	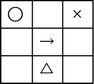	—
2	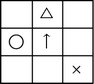	$\overset{{\text{90}}^{{}^{\circ}}\text{ to the left}}{\to }$	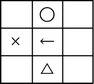	TTU	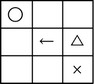	UU
3	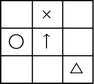	$\overset{{\text{180}}^{{}^{\circ}}\text{ to the right}}{\to }$	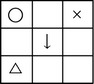	RRU	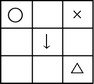	RR
4	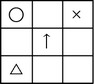	$\overset{{\text{180}}^{{}^{\circ}}\text{ to the left}}{\to }$	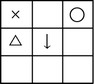	TTU	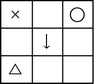	TT
5	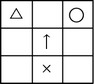	$\overset{{\text{90}}^{{}^{\circ}}\text{ to the right}}{\to }$	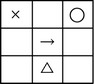	TT	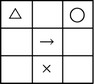	—
6	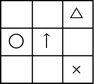	$\overset{{\text{90}}^{{}^{\circ}}\text{ to the left}}{\to }$	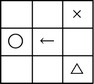	TT	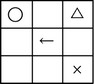	U
7	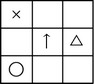	$\overset{{\text{180}}^{{}^{\circ}}\text{ to the right}}{\to }$	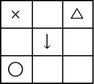	U	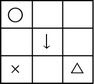	TTU
8	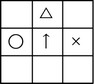	$\overset{{\text{180}}^{{}^{\circ}}\text{ to the left}}{\to }$	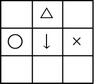	—	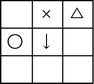	TU
9	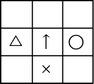	$\overset{{\text{90}}^{{}^{\circ}}\text{ to the right}}{\to }$	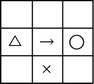	—	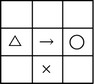	—
10	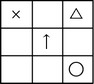	$\overset{{\text{90}}^{{}^{\circ}}\text{ to the left}}{\to }$	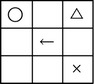	TT	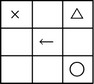	—

CPT-B score				14/30		18/30
Total errors				10T, 2R, and 4U		5T, 2R, and 5U

An arrow indicates the front of the subject.

T = transposition; R = rotational offset; and U = uncategorized errors.

**Table 3:** Comparison of CPT-B performance between the case and normal controls.

	Case	Controls (*n* = 11)	Statistics
Total errors/total CPT-B scores	28/32	69/261	*p* < 0.001^a^
Profile of error types	15T, 4R, and 9U	33T, 11R, and 25U	ns^b^

T = transposition; R = rotational offset; and U = uncategorized errors. ^a^Calculated by *χ* ^2^ test using a 2 × 2 contingency table. ^b^Calculated by *χ* ^2^ test using a 3 × 2 contingency table. ns = not significant.
